# Partial Clipping Occlusion Including Rupture Point Is an Effective Strategy for Ruptured Giant Fusiform Basilar Artery Aneurysm: A Technical Case Report

**DOI:** 10.3389/fneur.2021.743654

**Published:** 2021-09-30

**Authors:** Tsuyoshi Izumo, Takashi Fujimoto, Yoichi Morofuji, Yohei Tateishi, Takayuki Matsuo

**Affiliations:** ^1^Department of Neurosurgery, Nagasaki University Graduate School of Biomedical Sciences, Nagasaki, Japan; ^2^Department of Clinical Neuroscience and Neurology, Nagasaki University Graduate School of Biomedical Sciences, Nagasaki, Japan

**Keywords:** fusiform aneurysm, basilar artery, clipping, neurosurgery, skull base approach

## Abstract

Treatment of fusiform basilar artery aneurysms is still challenging today. The authors present a case of a patient with a ruptured giant fusiform basilar artery aneurysm successfully treated by clipping occlusion of the rupture point. A 62-year-old man suddenly fell into a coma due to subarachnoid hemorrhage (SAH) with a ruptured giant fusiform basilar artery aneurysm with a bleb on the right shoulder. We considered treating the lesion with stent-assisted coil embolization because of the aneurysm's shape, but we had to give up because stents were off-label in the acute phase SAH in our country. Instead, we successfully performed clipping surgery to partially occlude the aneurysm, including the rupture point via the anterior transpetrosal approach. His postoperative course was uneventful, without rerupture of the aneurysm, and his conscious level tended to improve. The postoperative imaging studies showed no complications and disappearance of the rupture point of the aneurysm. Although direct surgery for the giant fusiform basilar artery aneurysms is one of the challenging operations, it is an essential and highly effective treatment as a last resort for complex aneurysms if other treatments are not available.

## Introduction

Treatment of basilar artery fusiform aneurysms is still challenging today. Direct surgery for this aneurysm is complicated because the aneurysms are close to the critical structures of the brain stem, cranial nerves, and the perforators that supply blood to them, and the approach requires complex skull base procedures. In addition, due to the shape of the aneurysm, complete occlusion by simple clipping surgery is often complicated. Parent artery occlusion following extracranial to intracranial bypass surgery is usually required in pursuit of curability (1, 2) Since it is a complex operation and the complication rate is high, its indication is limited. The development of endovascular treatment for cerebral aneurysms has been remarkable in recent years. Several treatment effects using a stent-assisted coil embolization and a flow diverter have been reported for basilar artery fusiform aneurysms (3, 4), In contrast, serious complications such as brainstem infarction have also been reported. Thus, we should pay special attention to their indications. The authors present a case of a patient with a ruptured giant fusiform basilar artery aneurysm successfully treated by clipping occlusion of the rupture point.

## Case Description

**Figure 1:**
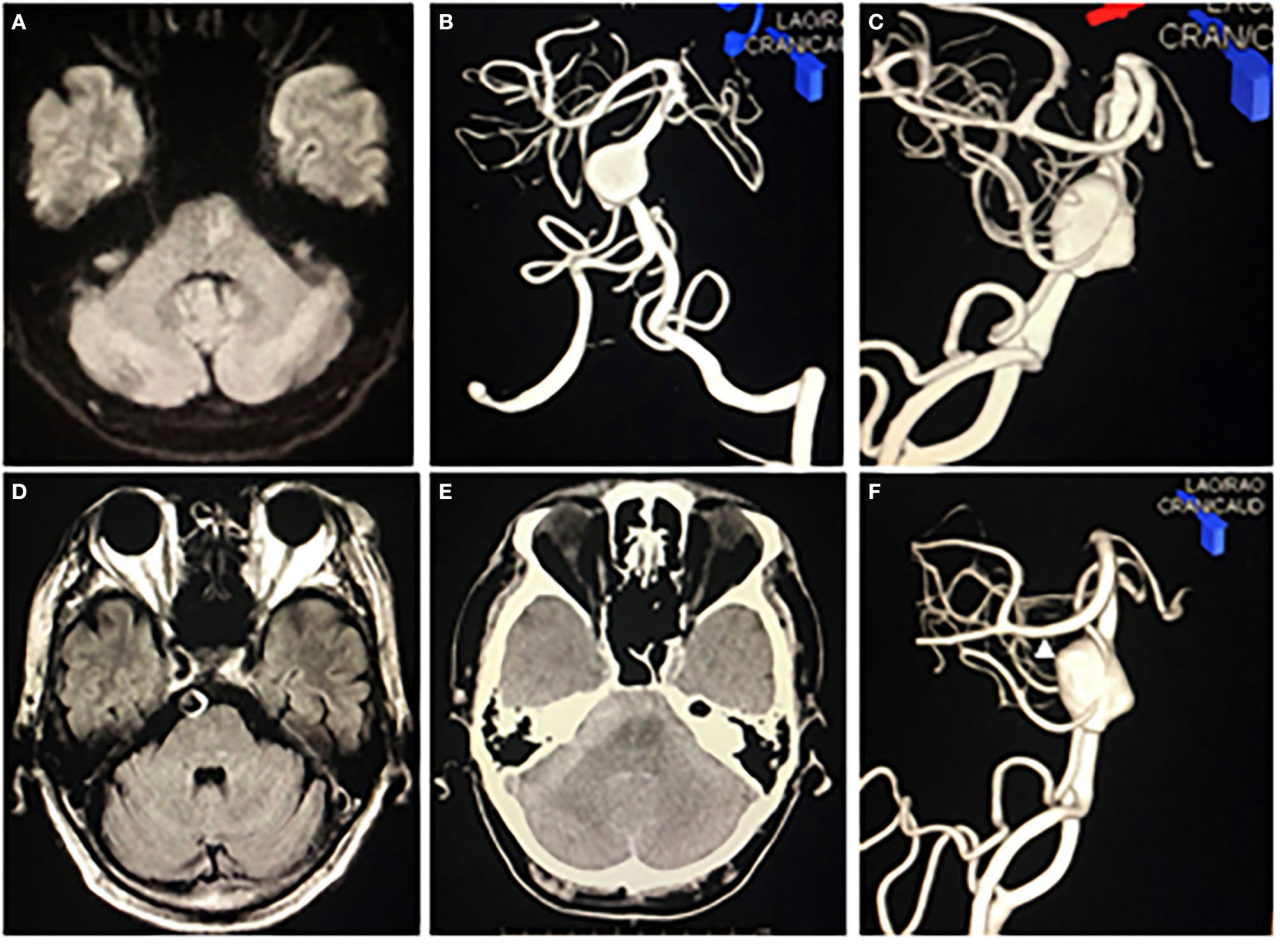
Preoperative imaging studies. **(A)** Diffusion-weighted image on MRI showed a small high-intensity lesion on left paramedian pons. **(B,C)** Anterior-posterior view **(B)** and right lateral view **(C)** of left vertebral artery multiplanar 3D rotational angiography revealed a giant fusiform basilar artery aneurysm. **(D)** A fluid-attenuated inversion recovery image on MRI revealed that the aneurysm was partially thrombosed. **(E)** CT showed diffuse subarachnoid hemorrhage incredibly thick in the right prepontine cistern. **(F)** Right lateral view of left vertebral artery multiplanar 3D rotational angiography revealed a bleb (white arrowhead) on the right shoulder of the fusiform basilar artery aneurysm.

A 62-year-old man presented to our department with left hemiplegia. His initial diagnosis was acute cerebral infarction (Figure 1A). He had been pointed out to have a partially thrombosed giant fusiform basilar artery aneurysm 3 months ago and we decided to follow up with imaging studies because the patient was asymptomatic and refused the treatment (Figures 1B–D). He suddenly fell into a coma on the third day of hospitalization. His plain computerized tomography (CT) of the head showed diffuse subarachnoid hemorrhage (SAH), which was relatively thick in the right anterior pontine cistern (Figure 1E). He underwent emergency ventricular drainage, and his level of consciousness improved to a Glasgow Coma Scale (GCS) of nine points. Subsequent digital subtraction angiography (DSA) revealed a newly formed bleb on the right shoulder of the previously noted fusiform basilar artery aneurysm (Figure 1F). From the distribution of SAH on CT and the findings of DSA, we assumed that the newly generated bleb was the rupture point this time. His preoperative modified Rankin Scale (mRS) score was 5.

## Operation Strategy

**Figure 2:**
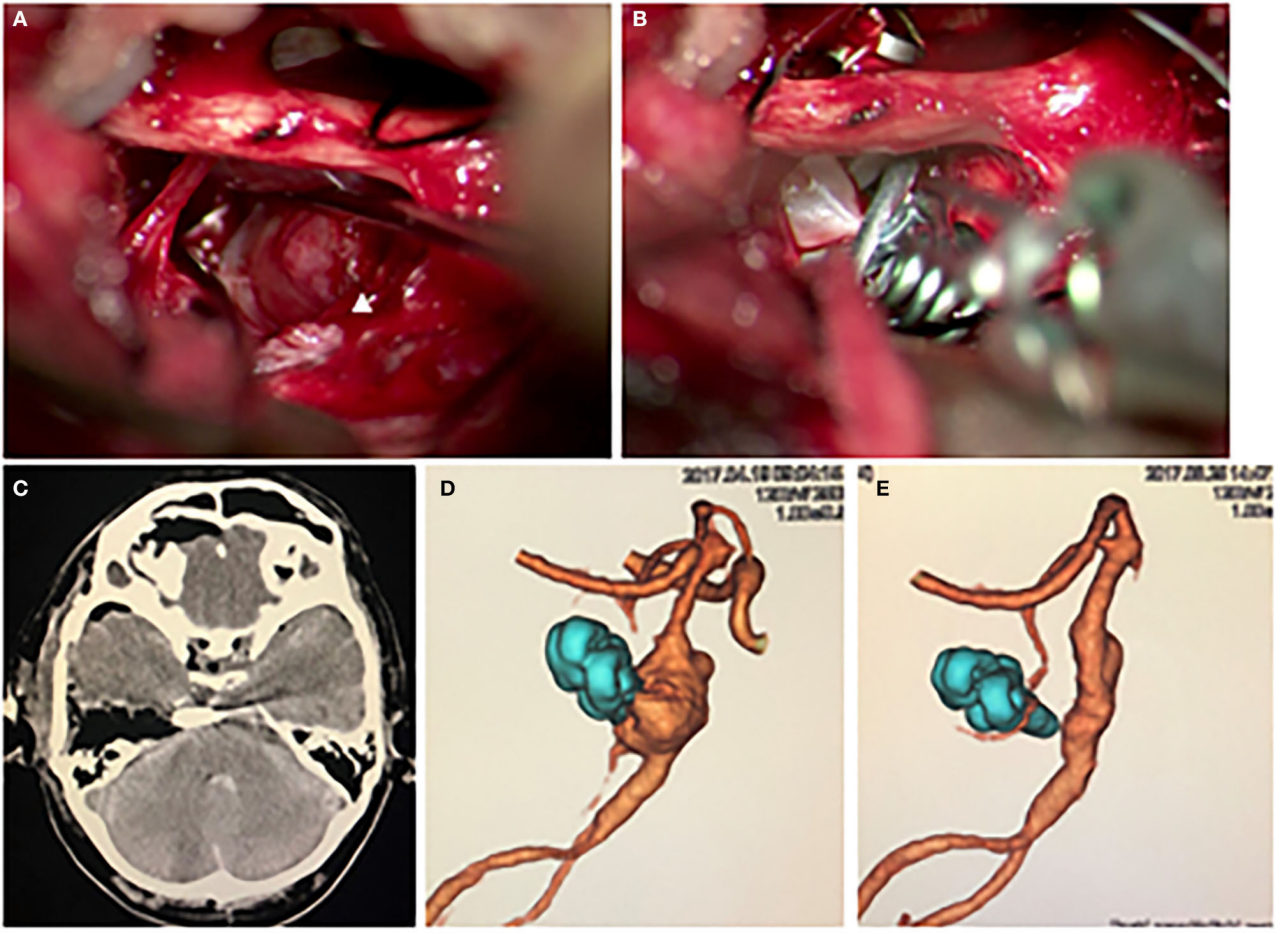
**(A,B)** The intraoperative findings. **(A)** The aneurysm was observed via the anterior transpetrosal approach. The white arrowhead indicates a bleb on the right shoulder of the aneurysm, which was covered by hematoma, indicating the bleb was the rupture point. **(B)** The aneurysm, including the bleb, was partially occluded with a combination clip using three clips. The photograph shows the third clip is being applied. **(C)** Postoperative CT showed no complication. **(D)** Postoperative 3D CT angiography showed the disappearance of the bleb. **(E)** A remarkable regression of the aneurysm was observed on 3D CT angiography 1 year after the surgery.

The patient underwent clipping surgery to prevent the re-rupture of the aneurysm. Since the aneurysm had a fusiform shape, we intended to occlude the aneurysm partially, including the rupture point. We placed the patient in a supine lateral position in which we rotated his head 90 degrees to the left. By performing right temporal craniotomy and extensive anterior pyramidal bone removal, we observed the aneurysm via the anterior transpetrosal approach. As expected by the preoperative imaging studies, we found the bleb on the right shoulder of the aneurysm to be the rupture point (Figure 2A). Careful inspection revealed that there was no perforator originating from the aneurysm. After a temporary occlusion of the basilar artery, we occluded the rupture point with three aneurysm clips in an angioplastical fashion to the basilar artery (Figure 2B). Intraoperative Doppler sonography and Indocyanine Green video angiography revealed occlusion of the rupture point of the aneurysm and good patency of the parent artery and branches.

## Postoperative Course

His postoperative plain CT showed no additional lesions, and 3-dimension CT angiography (3D-CTA) revealed a disappearance of the bleb of the fusiform aneurysm (Figures 2C,D). Although preoperatively noted his left hemiplegia persisted, his consciousness disturbance level gradually improved to a GCS of 14 points during the course without rerupture of the aneurysm. On the 31st day after the onset of SAH, he was transferred to a rehabilitation hospital with a mRS score of 4. One year after the surgery, his 3D-CTA revealed a remarkable shrinkage of the aneurysm (Figure 2E). We obtained informed consent for the publication from the patient and his family.

## Discussion

Fusiform basilar artery aneurysms are a rare entity with a prevalence report of 0.17–5.8% (4–6). The aneurysms are usually asymptomatic, but once they become symptomatic, they induce severe conditions such as consciousness disturbance, hemiplegia, dysphagia, ataxia, and death due to mass effect by enlargement, SAH due to rupture, and acute spontaneous occlusion (7–9). The exact annual rupture rate of the giant fusiform basilar artery aneurysms is currently unclear, Nakatomi et al. (2) reported that 11 patients of vertebrobasilar giant fusiform aneurysm did not undergo surgery, of whom 10 died. Thus, it is crucial to consider interventions for rupture prevention for the lesions.

No standard treatment for the giant fusiform basilar artery aneurysms has been established to date due to the rarity of the disease. However, endovascular treatment has been increasingly selected for the aneurysms in recent years. In particular, treatment by stent-assisted coil embolization is often chosen because of the shape of the aneurysm. This procedure's post-treatment complete occlusion rate for the aneurysms is high, with reports of 83.3–85% (10, 11). On the other hand, the complication rate associated with this procedure was relatively high, with reports of morbidity and mortality of 7.8% (12). There have been reports of cases of SAH due to rupture in the early postoperative period, and the recurrence rate is relatively high. Thus, caution should be paid in its indication (13).

Flow diversion (FD) for fusiform basilar artery aneurysms has become a promising treatment option. Initial reports with FD for fusiform basilar artery aneurysms were mixed with poor outcomes. But the FD treatment has been recently reported to have a good result with long-term aneurysm occlusion rate in many patients when FD is used in carefully selected patients (4, 14). On the other hand, there are reports of acute occlusion of the basilar artery and brain stem perforator occlusion after FD treatment (15, 16). These cause significant postoperative mortality and morbidity. Since hypercoagulable state occurs in the acute phase of SAH, these ischemic complications are likely to occur. Thus, the indication of FD for ruptured fusiform basilar artery aneurysms should be restricted to otherwise untreatable lesions (15).

Direct surgery for giant fusiform basilar artery aneurysms is still challenging today. Due to the aneurysm's shape, direct clipping is usually tricky, and parent artery occlusion following bypass surgery is often selected (1, 2, 17, 18). According to a review by Sughrue et al., direct surgery for giant aneurysms has a higher complete occlusion rate and lower retreatment and rebleeding rated than endovascular treatment but has relatively high morbidity. Nakatomi et al. (2) reported the surgical results of giant fusiform basilar artery aneurysms by various methods, including bypass surgery; a good prognosis was achieved in about 25% of the patients. Although this was better than the conservative treatment groups, the result indicates that the direct surgery for this aneurysm is still of great difficulty even with the latest technique.

The hybrid approach, the combination of the direct surgery and endovascular technique, is promising for the giant fusiform basilar artery aneurysms. One of the reasons why basilar aneurysms are challenging to clip is the difficulty of obtaining proximal control. Fortunately, we could get the bilateral distal vertebral artery for proximal control during the surgery. But endovascular balloon occlusion of basilar artery for proximal control during the direct surgery is a highly valuable procedure for the safety (19).

The authors present a case of a patient with a ruptured giant fusiform basilar artery aneurysm successfully treated by partial clipping occlusion of the aneurysm, including the rupture point without postoperative re-rupture of the aneurysm. In our country, neither stent nor FD is not available for the patient because each device is off-label for acute-stage ruptured aneurysm patients. Thus, direct surgical treatment was a last resort intervention for preventing the re-rupture of the aneurysm.

Achieving complete occlusion in brain aneurysm clipping is crucial from the perspective of rupture and recurrence prevention. Obermueller et al. (20) reported post clipping broad-based remnants are at a higher risk of regrowth. And postoperative regrowth is at a higher risk of rupture of the aneurysm (21). On the other hand, Nomura et al. (22) reported that acute partial embolization of the ruptured aneurysm was adequate for preventing subsequent rerupture. The surgery we performed for this case also has a policy of achieving partial occlusion using clips. This policy could prevent the re-rupture of the aneurysm in the acute phase of SAH even more than when using coils. Moreover, the aneurysm showed remarkable shrinkage on the imaging study 1 year after the surgery. Dissecting brain aneurysms have a higher frequency of spontaneous regression during follow-up than saccular aneurysms. It has been reported that the natural repair process causes this phenomenon (23). The mechanism is presumed to be that the mural thrombus induces vascular remodeling. Since the patient had a partially thrombosed aneurysm, the mural thrombus and clipping reconstruction of the basilar artery also yielded the vascular remodeling, leading to the remarkable regression of the aneurysm.

## Conclusion

The authors present a patient with a ruptured partially thrombosed giant fusiform basilar artery aneurysm successfully treated by partial clipping occlusion of the aneurysm, including the rupture point. Although direct surgery for the giant fusiform basilar artery aneurysms is one of the challenging operations, it is an essential and highly effective treatment as a last resort for complex aneurysms if other treatments are not available.

## Data Availability Statement

The original contributions presented in the study are included in the article/supplementary material, further inquiries can be directed to the corresponding author.

## Author Contributions

TI: conceptualization, methodology, and writing—original draft preparation. TI and YM: investigation. TI, TF, YM, and YT: writing—review and editing. TM: supervision. TI and TM: funding acquisition. All authors have read and agreed to the published version of the manuscript.

## Funding

This study was funded by Grants-in-Aid for Scientific Research (C) 18K08973 (to TI), (C) 21K09180 (to TI), and (C) 21K09129 (to TM).

## Conflict of Interest

The authors declare that the research was conducted in the absence of any commercial or financial relationships that could be construed as a potential conflict of interest.

## Publisher's Note

All claims expressed in this article are solely those of the authors and do not necessarily represent those of their affiliated organizations, or those of the publisher, the editors and the reviewers. Any product that may be evaluated in this article, or claim that may be made by its manufacturer, is not guaranteed or endorsed by the publisher.
